# The Costs and Cost-Effectiveness of Mass Treatment for Intestinal Nematode Worm Infections Using Different Treatment Thresholds

**DOI:** 10.1371/journal.pntd.0000402

**Published:** 2009-03-31

**Authors:** Andrew Hall, Sue Horton, Nilanthi de Silva

**Affiliations:** 1 Centre for Public Health Nutrition, University of Westminster, London, United Kingdom; 2 Micronutrient Initiative and School of Business & Economics, Wilfrid Laurier University, Waterloo, Ontario, Canada; 3 Department of Parasitology, Faculty of Medicine, University of Kelaniya, Ragama, Sri Lanka; Universidad Peruana Cayetano Heredia, Peru

## Abstract

**Background:**

It is estimated that almost a half of all of people living in developing countries today are infected with roundworms, hookworms, or whipworms or combinations of these types of intestinal nematode worms. They can all be treated using safe, effective, and inexpensive single-dose generic drugs costing as little as USD 0.03 per person treated when bought in bulk. The disease caused by intestinal nematodes is strongly related to the number of worms in the gut, and it is typical to find that worms tend to be aggregated or clumped in their distribution so that <20% of people may harbour >80% of all worms. This clumping of worms is greatest when the prevalence is low. When the prevalence rises above 50%, the mean worm burden increases exponentially, worms are less clumped, and more people are likely to have moderate to heavy infections and may be diseased. Children are most at risk. For these reasons, the World Health Organization (WHO) currently recommends mass treatment of children ≥1 year old without prior diagnosis when the prevalence is ≥20% and treatment twice a year when the prevalence is ≥50%.

**Methods and Findings:**

The risk of moderate to heavy infections with intestinal nematodes was estimated by applying the negative binomial probability distribution, then the drug cost of treating diseased individuals was calculated based on different threshold numbers of worms. Based on this cost analysis, a new three-tier treatment regime is proposed: if the combined prevalence is >40%, treat all children once a year; >60% treat twice a year; and >80% treat three times a year. Using average data on drug and delivery costs of USD 0.15 to treat a school-age child and USD 0.25 to treat a pre-school child (with provisos) the cost of treating children aged 2–14 years was calculated for 105 low- and low-middle-income countries and for constituent regions of India and China based on estimates of the combined prevalence of intestinal nematode worms therein. The annual cost of the three-tier threshold was estimated to be USD 224 million compared with USD 276 million when the current WHO recommendations for mass treatment were applied.

**Conclusion:**

The three-tier treatment thresholds were less expensive and more effective as they allocated a greater proportion of expenditures to treating infected individuals when compared with the WHO thresholds (73% compared with 61%) and treated a larger proportion of individuals with moderate to heavy worm burdens, arbitrarily defined as more than 10 worms per person (31% compared with 21%).

## Author Summary

Almost one in every two people in the developing world is infected with one or more types of intestinal nematode worms. When fewer than 50% of people are infected, most carry only a few worms; but when more than 50% are infected, the number carrying moderate to heavy numbers increases markedly, as does the risk of disease. The WHO recommends annual mass deworming of children when 20% or more are infected and twice a year if 50% or more are infected. We estimated the cost of this to treat children with 10+ worms, an arbitrary moderate to heavy infection. We concluded that it is not cost-effective to mass treat children when fewer than 40% are infected because the majority are uninfected and few are likely be diseased. We propose annual treatment when 40% or more children are infected, twice a year at 60%, and three times a year at 80% or more. This would cost USD 224 million annually to treat all children aged 2–14 years in 107 developing countries compared with USD 276 million using current WHO guidelines. The new three-tier guidelines also treat a larger proportion of infected children and treat children with moderate to heavy worm burdens more often.

## Introduction

In 2008 the Copenhagen Consensus ranked five nutritional interventions among the top ten of more than 40 proposals to answer the question: what would be the best ways of advancing human welfare globally? [1] Deworming and other nutrition programmes in school were ranked as the sixth best intervention overall when considering their potential benefit to cost ratio, anticipated feasibility and sustainability [1]. As drugs to treat worms are safe and inexpensive, it is feasible to give periodic mass treatment without the prior diagnosis of individual infections. However the cost, benefits and sustainability of mass treatment depend on the prevalence of infection used to decide whether and how often to give treatment, and on the proportion of infected individuals who have disease.

This paper has three sections. First, we introduce some of the key factors that affect how the disease caused by the three main types of intestinal nematode worms can be controlled. Second, we estimate the costs of treating people by mass chemotherapy depending on the proportion infected and on the number of worms that might cause disease. To do this we use data on the distribution among human hosts of the large roundworm, *Ascaris lumbricoides*. We then propose some new thresholds for giving mass treatment based on the cost per diseased person treated and compare them with the current World Health Organization (WHO) thresholds. We calculate the costs of deworming children from the ages of 2 to 14 y in the developing world using national and sub-national estimates of the prevalence of combined infections with any species of intestinal nematode worm. We conclude by discussing the implications of our analysis.

Intestinal worms number among the most common infections of people in the developing world today. It has been estimated that 1.22 billion people in low, lower-middle and upper-middle income economies, or 26% of their population, are infected with *Ascaris lumbricoides*, 0.80 billion (17%) with *Trichuris trichiura* and 0.74 billion (15%) with either or both of the two main species of hookworm, *Ancylostoma duodenale* or *Necator americanus* [2]. (These estimates exclude the former countries in the Commonwealth of Independent States for which prevalence data are not available). From these prevalences it has been estimated that about 48% of the population of these countries, or some 2.3 billion people, are likely to be infected with at least one of these species of intestinal nematode worms [3] (which assumes independence between species in the probability of infection, an assumption that is discussed further in the next section). The usual method to diagnose infections is to see the characteristic eggs of worms in faeces [4]. But the presence of eggs is a poor indicator of the risk of disease and of the impact of treatment because only two worms – a male and a female – are necessary to produce eggs, and two worms are very unlikely to cause disease. The risk of being diseased depends principally on the species and number of worms in the gut, as well as on the site in the gut in which the worms live, the mechanism by which worms feed, the duration of infection, the inflammatory and immune responses to infection, and the size, age and current health of the infected person [3].

**Figure 1:**
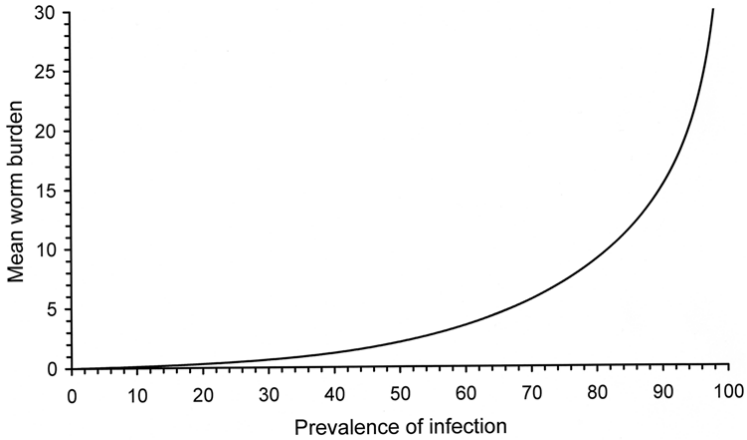
The relationship between the prevalence of infection with an intestinal nematode worm, in this case *Ascaris lumbricoides*, and the mean worm burden estimated by applying the negative binomial distribution using a clumping parameter (k) that varies linearly with the mean worm burden (see ref. [6]).

In simple numerical terms disease due to each species of intestinal nematode worms tends to be associated with moderate to heavy worm burdens, but there is no accepted number of worms that defines moderate to heavy infections for each species. Worms do not multiply within their host and each worm is the result of exposure to a fertilised and mature egg or larva. It is typical to find that worms are not randomly or evenly distributed between hosts and that more than 80% of all worms are present in less than 20% of all hosts [5]. This aggregated or clumped distribution of worms in a small proportion of hosts is best described empirically by the negative binomial probability distribution [5]. Observations of the numbers of *A.lumbricoides*, the worm most commonly studied, show that the degree of aggregation of worms among human hosts is greatest when the mean worm burden is low [6]. As the force of infection increases and the prevalence rises, the mean worm burden increases in a strikingly non-linear way (Figure 1) such that the degree of aggregation decreases and a larger proportion of individuals have moderate to heavy infections [7],[8].

Because worms are most aggregated when prevalence is low, at a prevalence of <50% only a small proportion of infected people will have disease due to a moderate to heavy worm burden and will therefore benefit from treatment. When the prevalence of infection is >50%, the cost-effectiveness of treatment rises because a larger proportion of the population have moderate to heavy worm burdens and are likely to be diseased.

The final important epidemiological characteristic of intestinal nematode worms is that surveys typically show that school-age children tend to harbour the heaviest infections with *Ascaris lumbricoides* and *Trichuris trichiura* [8],[9] and are the group in the population most likely to suffer from disease. As it is relatively easy to deliver treatments to children through the educational system, if they are enrolled, this can serve to minimise delivery costs [10] as well as having consequences for both health and education of children, two major benefits of treatment [11].

Mass treatment of children for worms is potentially cost-effective for number of reasons. First, when the prevalence of infection is greater than 50% the purpose of diagnosis becomes more to identify uninfected individuals to exclude from treatment than to identify those to treat. Second, the cost of the drugs to treat worms is measured in US cents while the cost of collecting and examining stool samples under a microscope to diagnose worms is measured in US dollars [12]. Third, the drugs to treat worms are very safe, meaning that there is no known harm for an uninfected person to be treated; they are effective to different degrees against all species of intestinal nematode [13]; and they can be given as a single, oral dose, which eliminates issues of compliance with treatment [14],[15]. Finally, as Figure 1 shows, the mean worm burden increases exponentially at prevalences above 50% and so, therefore, does the risk of disease [7].

As reinfection with intestinal worms can occur immediately after treatment, the aim of deworming is first, to reduce the number of worms substantially and eliminate disease, and then to repeat treatment often enough to prevent moderate to heavy loads from being re-accumulated [16].

In 1996 a WHO informal consultation endorsed a recommendation that a combined prevalence of any species of intestinal nematode worms of ≥50% was sufficient to warrant mass treatment of school-age children [17]. The WHO then developed a complicated strategy to decide how often to give mass treatment based on three categories of prevalence and the proportion of moderate to heavy infections based on arbitrary egg counts for each separate species [16]. This required an estimate of the concentration of eggs in faeces, which is not easy to do in a small rural hospital laboratory unless the necessary materials are supplied. The fecundity of worms, which largely governs the concentration of eggs in faeces, has also been shown to be highly variable between worms in different countries, for *A.lumbricoides* at least [18]. The practicability and the biological basis of this recommendation were therefore weak.

In 2006 another WHO informal consultation endorsed a reduction in the threshold for mass treatment in a simplified strategy called “preventive chemotherapy” [19]. If 20% to <50% of children are infected with intestinal nematode worms, then mass treatment is recommended for all school-age children once a year; if ≥50% of children are infected, then mass treatment is recommended twice a year [19]. Provision was also made for treatment three times a year if the prevalence of infection was ≥50% and if resources were available, but no different or higher threshold was specified [19]. The complicated strategy based on separate egg counts for different species was effectively superseded.

Mass deworming was promoted globally in 2001 when the 54^th^ World Health Assembly adopted a resolution to deworm at least 75% of all school-age children at least once a year in countries where intestinal worms were endemic [20], which is most of the developing world [2]. The most recent data available for 64 (49%) of the 130 countries in which worms are endemic indicated that coverage of schoolchildren was 22% [21], suggesting that the numbers presented in the most recent global estimates of the prevalence of infection [2] may not have changed significantly. Data for preschool children from 51 countries (39%) indicate that coverage has reached 55% [21].

## Methods

The cost of giving mass treatment with an anthelmintic drug was estimated in three ways: as the cost per person treated, as the cost per infected person treated, and as the cost per moderately-to-heavily infected person treated. The calculations were based on a drug cost of USD 0.03 per person, a typical price for a generic anthelmintic when bought in moderate quantities in a developing country.

The proportion of people who are infected is usually estimated by a survey to determine which people in a sample of the population have the eggs of worms in their faeces. The proportion of people who are moderately or heavily infected depends on two factors: how worms are distributed between individuals within a population and the threshold number of worms used to classify a worm load as a moderate infection.

The distribution of worms between hosts can usually be described empirically by the negative binomial probability distribution in which the prevalence *p* = 1−(1+M/*k*)^−*k*^ where M is the mean worm burden and *k* is a clumping parameter. Values of *k* are typically <1.0 for intestinal nematode worms [5] and reflect the observation that most worms tend to be aggregated in a small proportion of all hosts. The negative binomial probability distribution was applied to estimate the proportion of individuals with more than any given threshold number of worms, as follows.

The observed linear relationship between the mean worm burden and the clumping parameter for *A.lumbricoides*, in which *k* = 0.334+0.0172 M [6], was used to estimate values of *k* for values of M in steps of roughly M/2, starting at M = 30, the highest mean worm burden ever reported [22]. These values of M and *k* were then used to estimate the proportion infected by applying the equation for the negative binomial distribution. Values of M were adjusted, which also altered *k*, to give proportions infected ranging from 20% to 95% in steps of 10% to 80% and then steps of 5% to 95%.

In the next stage the values of *k* and M for each value of prevalence between 20% and 95% were entered into the negative binomial function (*pnbinom*) of R statistical software version 2.7.2 [23] to estimate the proportion of people in any population who have more than 5, 10, 15 or 20 worms, arbitrary thresholds which could be applied to classify a worm burden as moderate or heavier. The final values of *p*, M and *k* used to estimate the proportions of moderately to heavily infected individuals are shown in Table 1.

**Table 1:** Estimates of the costs of treating infections with *Ascaris lumbricoides* using a drug costing USD 0.03 per dose calculated in three ways: per person treated, per infected person treated, and per diseased person treated defined using four different thresholds of worm burden.

							Costs in USD per					
Proportion infected (*p*)	Mean burden (M)	Clumping parameter (*k*)^a^	Proportion infected with				Person treated	Infected person treated	Diseased person treated if disease			
			≥5 worms	≥10 worms	≥15 worms	≥20 worms			≥5 worms	≥10 worms	≥15 worms	≥20 worms
0.95	30.0	0.850	0.8158	0.6860	0.5817	0.4956	0.03	0.03	0.04	0.04	0.05	0.06
0.90	20.0	0.678	0.6988	0.5430	0.4322	0.3483	0.03	0.04	0.04	0.06	0.07	0.09
0.85	14.5	0.583	0.6032	0.4377	0.3295	0.2526	0.03	0.04	0.05	0.07	0.09	0.12
0.80	11.0	0.523	0.5241	0.3563	0.2539	0.1852	0.03	0.04	0.06	0.08	0.12	0.16
0.70	6.4	0.442	0.3783	0.2188	0.1354	0.0865	0.03	0.04	0.08	0.14	0.22	0.35
0.60	3.6	0.396	0.2530	0.1159	0.0580	0.0302	0.03	0.05	0.12	0.26	0.52	0.99
0.50	2.0	0.368	0.1456	0.0460	0.0162	0.0060	0.03	0.06	0.21	0.65	1.85	5.01
0.40	1.2	0.354	0.0766	0.0149	0.0033	0.0008	0.03	0.07	0.39	2.01	9.09	38.88
0.30	0.6	0.345	0.0222	0.0016	0.0001	0.0000	0.03	0.10	1.35	18.80	230.81	2,658.33
0.20	0.3	0.339	0.0041	0.0001	0.0000	0.0000	0.03	0.15	7.35	485.68	27,335.62	1,435,480.19

The method used to calculate the parameters is described in the text. Some values for the proportion infected were <0.0001. Adapted from ref [48].

a *k* = a+bM in which a = 0.334 and b = 0.0172 [7].

Although this analysis is based on data for a single species of worm, *Ascaris lumbricoides*, it may apply to other species of intestinal worms because the distribution of disease for any species is driven largely by the extent to which worms are aggregated in a few hosts, so the clumping parameter *k* is of great importance. As values of *k* range from 0.03–0.6 for hookworms and from 0.2–0.4 for whipworms [5], they indicate a greater degree of aggregation of these species than for roundworms, values for which range from 0.3–0.9 [5]. Similarly the coefficients in the equation linking *k* and the worm burden M in Table 1 have been estimated for roundworm, but are not known so well for the other species. Hence the numerical values used in this analysis are not necessarily a perfect guide to cases of infection with whipworm or hookworm, or indeed mixed infections with more than one kind of intestinal worm. However the main point, that worms are most highly aggregated at low prevalences of infection, is likely to hold true for all species.

As there are no existing classifications of the numbers of worms of any species that cause disease they can only be guessed at based on a knowledge of the size of worms or the estimated effects of individual worms. For example, assuming an equal sex ratio, 5 *A.lumbricoides* weigh about 12 g, 10 worms weigh about 25 g, 15 worms weigh 35 g and 20 worms weigh about 45 g, which is about 0.3% of the body weight of an underweight 6-year old child weighing 15 kg [24]. The energy requirements of a worm burden such as this are thought to be relatively small in comparison with the host, but worms may have more important effects on absorption and appetite [3]. For the hookworm species *A.duodenale*, 5 worms are estimated to cause a blood loss of 1 ml/day, 10 worms 2 ml/day, 15 worms 3 ml/day and 20 worms 4 ml/day; the same figures for the other hookworm species, *N.americanus*, are 0.2, 0.4, 0.6 and 0.8 ml/day of blood a day, as the two species differ in the volume of blood loss that they cause [25]. But as both species now occur together widely throughout the world, mixed infections are very likely [26]. The loss of blood caused by the same number of whipworms is estimated to be 0.02, 0.05, 0.07 and 0.1 ml/day of blood [27] although the inflammatory response to the head of the worms embedded in tissues may be more important.

For the purposes of calculating the number of moderately to heavily infected individuals and to estimate the costs of treating them on the assumption that they are diseased, thresholds burdens of ≥5, ≥10, ≥15 and ≥20 worms were used to estimate the cost of treating each diseased person. This analysis is presented in Table 1 and shows that when an arbitrary threshold of 10 worms is used to define a moderate infection, a cost-effective threshold for giving mass treatment of less than USD 1 per diseased person treated lies between a prevalence of 40% and 50%. We therefore propose the following new thresholds for mass treatment, which we call the three-tier treatment thresholds:

- when the prevalence is 40% to <60%, mass treat once a year;
- when the prevalence is 60% to <80%, mass treat twice a year;
- when the prevalence is 80% to 100%, mass treat three times a year.

These thresholds are both simple and evenly incremental and they apply treatment more often in circumstances in which the risk of moderate to heavy infections is highest.

When the prevalence is <40% we propose that only underweight, wasted or anaemic children should be treated as a matter of course or that internationally recommended guidelines should be followed, such as those for the Integrated Management of Childhood Illness.

As the estimates of cost presented in Table 1 do not include the cost of delivering treatments, the second stage of the analysis was to estimate and compare the cost of delivering an anthelmintic drug costing USD 0.03 per dose to all children in the developing world by applying either the WHO guidelines or the new three-tier guidelines. To do this estimates were made of the prevalence of infection with any type of intestinal nematode worms in 107 developing countries, of the numbers of children to be given mass treatment in the same developing countries, and of the costs of delivering treatments to all pre-school and school-age children, as follows.

As most surveys report the prevalence of each worm species separately, rather than as a combined prevalence of intestinal nematode worms, the combined prevalence in each of 107 developing countries was estimated using data from ref. [2] based on the assumption that the probability of infection with one species was independent of infection with any other. For example, if the prevalence of infection with *A.lumbricoides* was 60% and the prevalence of *T.trichiura* was 40%, then the prevalence of joint infections was estimated to be 0.6×0.4 = 0.24. Thus the proportion infected with either or both species was estimated to be: (0.6–0.24)+(0.4–0.24)+0.24 = 0.76. A preliminary analysis of data from 38 surveys in 16 countries which reported the combined prevalence of any species of intestinal nematode as well as the individual prevalence of each species, gave a correlation between the observed prevalence and the calculated combined prevalence using this method of 0.99 (*P*<0.0001) (data not reported).

Using these survey data the combined prevalence of all three main types of intestinal nematode worms was estimated for most developing countries. Because China and India contain a large proportion of the world's children, sub-national data on prevalence taken from the 2^nd^ national survey were used for China's provinces, autonomous regions or municipalities [28], and data from a previous analysis were used for India's states or union territories [2]. The data were then mapped.

The number of children aged 2–14 years in the same developing countries was estimated for each county and for the separate provinces of China and states of India using United Nations population data [29] and data from the censuses of China [30] and India [31] by applying World Health Organization life tables [32]. The numbers used in these calculations are given in Tables S1 and S2.

Estimates of the cost of delivering a single dose drug to treat intestinal nematode worms were derived from published reports, summarised in Table 2. A cost per round of treatment of USD 0.25 for each pre-school child and USD 0.15 per school-age child was used in the analysis. These costs assume that a generic drug costing USD 0.03 is applied and that deworming is combined with another intervention for preschool children or is given in schools with another treatment such as praziquantel, thus apportioning the distribution and delivery costs with at least one other intervention.

**Table 2:** Estimates of the cost of delivering single dose treatments with albendazole (ALB) or praziquantel (PZQ) to treat pre-school and school children for infections with intestinal nematode worms or *Schistosoma* spp respectively.

Country	Group	Treatment	Delivery cost per child per dose	Reference
Ghana	Schoolchildren	Albendazole, Praziquantel	USD 0.07 (ALB), USD 1.19 (PZQ) Including costs of volunteers time	[10]
Tanzania	Schoolchildren	Albendazole, Praziquantel	USD 0.04 (ALB), USD 0.30 (PZQ) Including costs of volunteers time	[10]
Uganda	Schoolchildren	Albendazole, Praziquantel (if schisto >30%), once/year	USD 0.54 (USD 0.32 excluding drug cost for PZQ)	[38]
Tanzania	Schoolchildren	Albendazole, Praziquantel (if schisto >50%), once/year	USD 0.23/round ALB, USD 0.79 PZQ	[49]
Ethiopia	Preschool children	Albendazole, Vitamin A	USD 0.57 incl also vitamin A (vitamin A & worm supply costs similar)	[50]

Note. All programs combined the distribution of drugs to treat intestinal nematodes with another intervention, either praziquantel for schoolchildren or vitamin A for preschoolers. The costs in stand-alone programmes would be higher, as distribution costs would not be shared with another programme. Although costs are only available for countries in Africa, these are considered to be a reasonable guide to costs in South, Southeast and East Asia. Costs in Latin America and the Caribbean are likely to be higher due to higher salary costs.

The combined prevalence of any intestinal nematode worm was then used to classify countries into those requiring none, one or two annual treatments in the first year based on the current WHO thresholds, or requiring none, one, two or three annual treatments in the first year using the three-tier treatment thresholds. The total cost was calculated as well as the proportion spent on treating infected people only and the proportion spent on treating people with 10 or more worms.

## Results

Table 1 shows the values of *p*, M and *k* used to estimate the proportions infected with between 5 or more to 20 or more *Ascaris lumbricoides* with the cost per person, per infected person and per diseased person, depending on the threshold number of worms used to define disease.

When the prevalence of infection approaches 100%, as it can in places where transmission is very intense, then the cost per infected person treated approaches the cost per person treated. However when the prevalence is <100% some uninfected people will be treated unnecessarily, so that when the prevalence is as low as 20%, the minimum prevalence at which the WHO recommend mass treatment [19], the cost of drugs per infected person treated is 5 times the cost per person treated (Table 1).

Table 1 also shows that when the prevalence is 20% only 0.004% of individuals may have ≥5 worms, a relatively low threshold at which disease might occur. When mass treatment is given at this threshold the drugs alone would cost USD 7.35 per person treated with 5 or more worms. For a threshold of 10 or more worms the cost is USD 485 per diseased person treated and when the threshold is increased to 15 or 20 worms, the cost is two orders of magnitude greater for each increase of 5 worms (Table 1).

**Figure 2:**
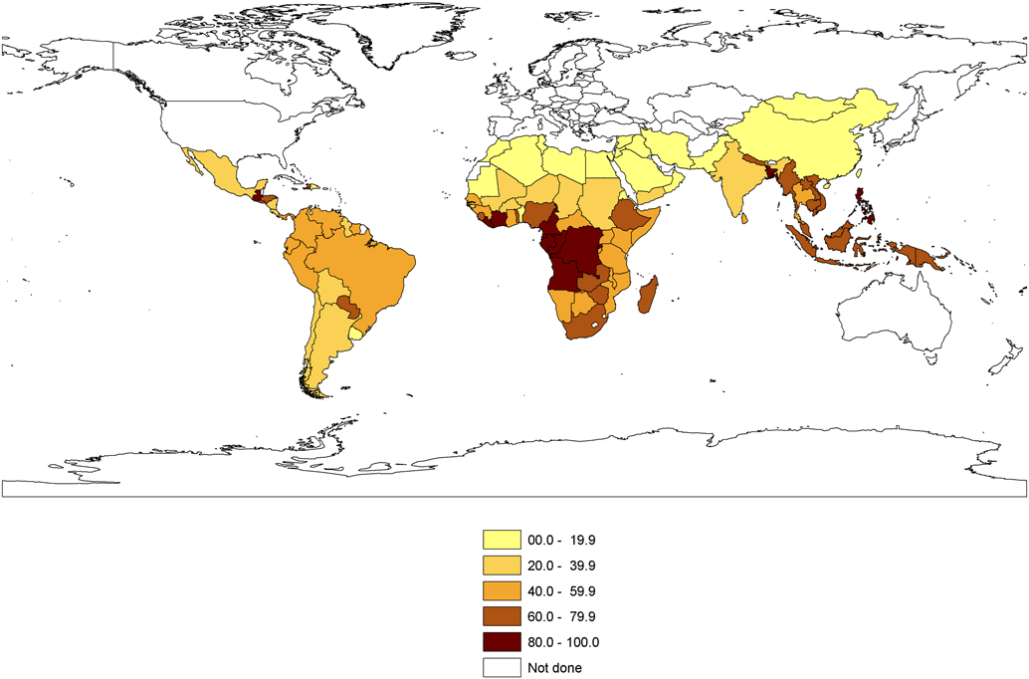
A map of the prevalence of infection with any species of intestinal nematode worms derived from data presented in ref [2] on the national prevalences of combined infections with *Ascaris lumbricoides*, *Trichuris trichiura* and the two hookworm species *Ancylostoma duodenale* and *Necator americanus*. The method of calculation is described in the text.

Figure 2 shows a map of the combined prevalence of infection with all three main types of intestinal nematode worms in 107 developing countries. Table 3 shows how these countries, the provinces of China and the states and territories of India are classified into six groups requiring none, one, two or three annual treatments in the first year using the current WHO thresholds and the new three-tier treatment thresholds.

**Table 3:** Classification of countries (see Table S1) and regions of India and China (see Table S2) by prevalence of infection and annual treatment frequency (x0, x1, x2 or x3 times) based on current WHO guidelines [19] and the new three-tier guidelines proposed here.

Group 1	*Group 2*	Group 3	*Group 4*	Group 5	*Group 6*
Prevalence: <20%	*Prevalence: 20–39%*	Prevalence: 40–49%	*Prevalence: 50–59%*	Prevalence: 60–79%	*Prevalence: ≥80%*
WHO: x0	*WHO: x1*	WHO: x1	*WHO: x2*	WHO: x2	*WHO: x2 (flexible)*
Three-tier: x0	*Three-tier: x0*	Three-tier: x1	*Three-tier: x1*	Three tier: x2	*Three tier: x3*
Algeria	Argentina	Botswana	Burundi	Cambodia	Angola
Benin	Bahamas	Brazil	Central African Rep.	El Salvador	Bangladesh
China (20 provinces)	Barbados	China (3 provinces)	China (1 province)	Ethiopia	Cameroon
Dominican Rep.	Bolivia	Colombia	Guinea	Haiti	Congo
Egypt	Chad	Ecuador	India (4 states)	Honduras	Congo DR
Eritrea	Chile	Ghana	Jamaica	India (2 states)	Cote d'Ivoire
India (15 states)	China (7 provinces)	India (5 states)	Kenya	Indonesia	Equatorial Guinea
Iran	Costa Rica	Malawi	Namibia	Laos	Fiji
Iraq	Grenada	Mozambique	Panama	Madagascar	Gabon
Jordan	Guinea-Bissau	Senegal	Peru	Malaysia	Guatemala
Lebanon	Guyana		Somalia	Maldives	Liberia
Libya	India (9 states)		Sri Lanka	Myanmar	Micronesia
Mauritania	Mali		Suriname	Nepal	Philippines
Mongolia	Mauritius		Tanzania	Nigeria	Rwanda
Morocco	Mexico		Thailand	Papua New Guinea	Samoa
Oman	Nicaragua		Uganda	Paraguay	Sao Tome and Principe
Pakistan	Niger		Venezuela	Sierra Leone	Solomon Islands
Puerto Rico	St. Lucia			South Africa	Tonga
Saudi Arabia	St. Vincent			The Gambia	Vanuatu
Syria	Sudan			Togo	
Trinidad & Tobago	Yemen			Vietnam	
Tunisia				Zambia	
Uruguay				Zimbabwe	

Columns 2, 4 and 6 with titles in italics represent differences in approach. Note: 10 developing countries were excluded due to lack of prevalence data (Afghanistan, Belize, Bhutan, Cape Verde, Comoros, Cuba, Djibouti, Lesotho, North Korea and Swaziland). Another 11 smaller countries did not have population age structure data available, and were also omitted: (American Samoa, Antigua, Cook Islands, Dominica,Kiribati, Marshall Islands, Nieu, Palau, Seychelles, St. Kitts, Tuvalu). The former Commonwealth of Independent States countries are also excluded due to lack of revalence data. Source: authors' calculations. Prevalence data are provided in Table S1 (national data) and Table S2 (subnational data for India and China).

Table 4 shows the total costs of applying treatments based on the classification of countries by group in Table 3. The new three-tier guidelines cost almost 20% less than the current WHO guidelines, at USD 224 m compared with USD 276 m. This is because although the new guidelines recommend fewer treatments when the prevalence is low, this is partially offset by more frequent treatment at higher prevalences, thus focussing resources in places where moderate to heavy infections are most likely [19].

**Table 4:** Annual costs of treating children aged 2–14 y by countries grouped by prevalence (see Table 3) and depending on the frequency of treatment given according to current WHO recommended thresholds [19] and the new three-tier thresholds proposed here.

Country group from Table 3	Prevalence of infection %	USD millions	USD millions
		Three-tier thresholds	WHO thresholds
Group 2	20–39	0	54.6
Group 3	40–49	23.7	23.7
Group 4	50–59	22.4	44.8
Group 5	60–79	102.8	102.8
Group 6	80–100	74.8	49.9
Total		223.7	275.8
% spent on initially-infected individuals		72.6%	61.1%
% spent on individuals initially infected with 10+ worms		31.0%	21.4%

Notes. The population estimates are for children aged 0–14 y from ref. [29] converted to age range 2–14 y using the best approximation possible taken from the WHO Life Tables [32] as follows. The number of person-years lived, _n_L_x_ was applied to obtain total person-years lived from zero to below the age of 15 y (_15_L_0_) and from this was subtracted the number of person-years lived below the age of 1, one quarter of the person-years lived between 1 and 5, and one fifth of the person-years lived between 10 and 15, to obtain the proportion of the under-15 population who are at least 2 years old and 14 y or under: i.e. _12_L_2_ = _15_L_0_−(_1_L_0_+0.25*_4_L_1_+0.2*_5_L_10_). The underlying data are provided in Table S1. Note that the estimates of percentages spent on initially-infected individuals, and those initially infected with 10+ worms, are based on static prevalence, and do not take into account declining prevalence with re-treatment. A dynamic model would be desirable.

Because of the different thresholds at which a single annual treatment is given, the new three-tier thresholds spent 73% of expenditure on treating infected individuals in the first year compared with 61% for the WHO guidelines (Table 4). This means that 27% and 39% of costs respectively were used to treat uninfected individuals, an inevitable consequence of giving mass treatment.

The new guideline also led to greater spending on treating moderately to heavily infected individuals. The proportion of expenditures on treating individuals with burdens of 10 or more worms was 31% for the three-tier guidelines compared with 21% for the WHO guidelines (Table 4).

The number of treatments in later years and hence the continuing costs will depend on the prevalence of infection and whether it is reduced to a lower threshold band. If so, the frequency of subsequent treatments can be reduced. To assess a change in prevalence will require data from small surveys before and after treatment, though the interval could be as infrequent as every two years, depending on local resources and capacity.

## Discussion

The analysis presented here indicates that a prevalence of infection with any species of intestinal nematode worm of 40% or more provides a cost-effective threshold at which to give mass treatment once a year. Because the proportion of moderate to heavy infections increases non-linearly with an increasing prevalence (Figure 1), higher thresholds of 60% and 80% provide a simple but epidemiologically sound and cost-effective basis on which to treat twice or three times a year, at least in the first 1–2 years of a programme. While models of reinfection indicate that it may be more important to achieve higher population coverage than more frequent treatment, [33] it is easier and less costly to achieve high coverage of children than adults. Giving treatments three times a year in places with a high initial prevalence will bring down mean worm burdens more quickly than treating only twice a year, while missing one of three treatments will achieve better annual coverage of any given individual than if one of only two treatments is missed, as the WHO recommend when the initial prevalence is greater than 50%. As children with moderate to heavy worm burdens tend to become moderately to heavily reinfected after treatment because of unknown factors that contribute to a predisposition to heavy infections [34]–[36] it may be better to specify three rather than two annual treatments in order to sustain low worm burdens in the face of rapid reinfection [37]. It has also been shown that over several cycles of treatment and reinfection with *A. lumbricoides*, at least two thirds of all individuals treated may become moderately heavily reinfected at least once [37] so that, over time, a large proportion will benefit from repeated treatment.

This analysis shows that when the costs of deworming drugs are calculated per individual treated it ignores that fact that not everyone is infected and underestimates the cost per infected person treated. Likewise, when the costs of deworming are given per infected individual treated it ignores the fact that not everyone has a significant worm burden and underestimates the cost per moderately or severely infected person treated.

The estimates of the cost of deworming drugs shown in Table 1 attempt to take into account the distribution of disease caused by intestinal nematode worms, which is affected mainly by the worm burden. But this depends on the epidemiological parameters used in the negative binomial distribution and the threshold burden at which disease is classified, which is arbitrary. But if the same biological factors that cause the aggregation of *A.lumbricoides* also apply to *T.trichiura*, *A. duodenale* and *N.americanus*, then it could be argued that if the disease they cause is additive rather than synergistic, then the threshold number of worms used in Table 1 could apply to the combined number of worms of any of these species to define disease. It would be worthwhile undertaking further research on the worm burdens of mixed infections, since these are the basis for treatment thresholds.

The analysis presented in Table 1 suggests that the cost of treating diseased people becomes uneconomically high when the prevalence is less than 40%. Cost-effectiveness data do not support the preventive chemotherapy approach of treating once per year for prevalence rates as low as 20%, unless an appropriate longitudinal model of reinfection and disease can be developed that would justify treatment.

The costs of delivering treatments will depend on local circumstances but tend to be an order of magnitude greater than the cost of the drugs. Delivery costs are typically around four times the cost of the drugs for schoolchildren and six times the cost for preschoolers (Table 2). Delivery costs may also vary with population density [38], probably because fixed delivery costs are divided by a larger denominator. The implication is that the marginal cost of increasing coverage is likely to increase as coverage also increases. Whether the marginal benefit also increases as coverage of treatment reaches remote populations will depend on whether worm burdens are higher in more remote areas, perhaps because of lack of access to sanitation for example, or lower, perhaps because of lower population density and lower transmission.

The coverage of school-age children will depend largely on enrolment rates which are improving as a result of global efforts to achieve one of the Millennium Development Goals, but will probably miss many non-enrolled children. But there is no reason why coverage of children who are enrolled and attending school should not approach 100%, especially if children who are absent from school are treated later. In any case, there are externalities in terms of treatment and its effects on reducing transmission that will also benefit those children who miss being treated. Mass treatment has been shown to lead to a reduced prevalence of infection among untreated people [39],[40].

As programmes to control both intestinal nematode worms and lymphatic filariasis using albendazole given with ivermectin or DEC have been implemented recently in parts of some countries, the national prevalence of intestinal nematode worms may have been reduced to some degree. This may mean that the aggregate costs for both sets of guidelines may be overestimated.

In order to improve cost-effectiveness it is recommended that small surveys of the prevalence of intestinal nematode worms be undertaken to establish the need for treatment and its frequency and then repeated every one to two years, depending on resources and capacity, to monitor prevalence and decide how often treatment should be given each year. Faecal egg counts should be estimated as a matter of good practice about 21 days after treatment to assess both the cure rate and the egg reduction rate in order to detect drug resistance [17]. However egg counts may not give a good estimate of the effect of treatment on worm loads if the fecundity of any remaining worms rises after treatment because of the partial removal of density dependent constraints on egg production [41]. Although such surveys increase costs modestly, they can increase cost-effectiveness by avoiding unwarranted treatments.

It is also important to state that deworming is a short to medium term intervention to control disease due to intestinal worms and that efforts should also be made in schools and communities to install, maintain and use sanitary latrines [42], to provide clean water and soap to remove worm eggs from contaminated hand before eating; and to promote healthy behaviours through health education [43]. These are the long term measures that will help to sustain a reduced prevalence of infection by keeping people and their faeces apart.

The estimated cost of the three-tier approach of over USD 224 million in the first year (USD 276 million using the WHO guidelines) to treat all children in the countries listed in Table 4 is a large expenditure, but it should decline annually as the prevalence goes down. However, the potential benefits in terms of improved child health and education are very large if a benefit∶cost ratio of 6∶1 is applied for preschool children [44], and are even higher for school-age children for whom the delivery costs are lower. One estimate is 60∶1, although this excludes the cost of hiring additional teachers if participation rates in education increase [40].

There are current programmes to treat schoolchildren supported by governments and NGOs in sub-Saharan Africa, the Indian sub-continent and Asia [45]. Some countries, such as Ethiopia, Bangladesh and Uganda, are giving mass treatment with albendazole or mebendazole to preschool children at the same time as they give vitamin A, for example during Child Health Days [46],[47]. Delivery costs may also be reduced if they can be split with other programmes, such as lymphatic filariasis control, which provides albendazole in addition to ivermectin or diethyl carbamazine. There is now a need to collect sub-national data, probably for regions or provinces in all countries in which worms are endemic, and to refine these calculations based on local prevalence data and local cost circumstances. This will provide accurate local and national estimates of the costs of treating a group of worms that make a major contribution to the burden of neglected tropical disease.

## Supporting Information

Table S1 Data for each country on the prevalence of infection with any of the main types of intestinal nematode worm, the population aged 0–15 years and the proportion aged 2–14 y, used for the classification of countries in Table 3 and the calculations in Table 4.
(0.12 MB DOC)

Table S2 Sub-national data for India (left) and China (right) on the prevalence of infection with any of the main types of intestinal nematode worm, the total population, and the population aged 2–14 y, used for the classification of states and territories of India and provinces, autonomous areas and municipalities of China in Table 3 and for the calculations in Table 4.
(0.10 MB DOC)

## References

[1] Copenhagen Consensus (2008). Copenhagen Consensus 2008 - Results.. http://www.copenhagenconsensus.com/Default.aspx?ID953.

[2] de Silva NR, Brooker S, Hotez PJ, Montresor A, Engels D (2003). Soil-transmitted helminth infections: updating the global picture.. Trends Parasitol.

[3] Hall A, Hewitt G, Tuffrey V, de Silva N (2008). A review and meta-analysis of the impact of intestinal worms on child growth and nutrition.. Matern Child Nutr.

[4] WHO (1991). Basic Laboratory Methods in Medical Parasitology.

[5] Anderson RM, May RM (1991). Infectious Diseases of Humans. Dynamics and Control.

[6] Guyatt HL, Bundy DA, Medley GF, Grenfell BT (1990). The relationship between the frequency distribution of *Ascaris lumbricoides* and the prevalence and intensity of infection in human communities.. Parasitology.

[7] Guyatt HL, Bundy DA (1991). Estimating prevalence of community morbidity due to intestinal helminths: prevalence of infection as an indicator of the prevalence of disease.. Trans R Soc Trop Med Hyg.

[8] Hall A, Anwar KS, Tomkins A, Rahman L (1999). The distribution of *Ascaris lumbricoides* in human hosts: a study of 1765 people in Bangladesh.. Trans R Soc Trop Med Hyg.

[9] Bundy DA, Cooper ES, Thompson DE, Anderson RM, Didier JM (1987). Age-related prevalence and intensity of *Trichuris trichiura* infection in a St. Lucian community.. Trans R Soc Trop Med Hyg.

[10] Partnership for Child Development (1999). The cost of large-scale school health programmes which deliver anthelmintics to children in Ghana and Tanzania.. Acta Trop.

[11] Bundy DA, Guyatt HL (1996). Schools for health: Focus on health, education and the school-age child.. Parasitol Today.

[12] Carabin H, Guyatt H, Engels D (2000). A comparative analysis of the cost-effectiveness of treatment based on parasitological and symptomatic screening for Schistosoma mansoni in Burundi.. Trop Med Int Health.

[13] de Silva N, Guyatt H, Bundy D (1997). Anthelmintics. A comparative review of their clinical pharmacology.. Drugs.

[14] WHO (1995). WHO Model Prescribing Information. Drugs Used in Parasitic Diseases.

[15] WHO (2005). World Health Organization Model Formulary.

[16] WHO (2002). Prevention and control of schistosomiasis and soil-transmitted helminthiasis.

[17] WHO (1996). Report of the WHO Informal Consultation on the use of chemotherapy for the control of morbidity due to soil-transmitted nematodes in humans.

[18] Hall A, Holland C (2000). Geographical variation in *Ascaris lumbricoides* fecundity and its implications for helminth control.. Parasitol Today.

[19] WHO (2006). Preventive chemotherapy in human helminthiasis.

[20] Assembly WH (2001). Schistosomiasis and soil-transmitted helminth infections. Fifty-fourth World Health Assembly WHO54.19.

[21] WHO (2008). Soil-transmitted helminthiasis: progress report on number of children treated with anthelmintic drugs.. Wkly Epidemiol Rec.

[22] Arfaa F, Ghadirian E (1977). Epidemiology and mass-treatment of ascariasis in six rural communities in central Iran.. Am J Trop Med Hyg.

[23] R Development Core Team (2007). R: a language and environment for statistical computing.

[24] WHO (1983). Measuring change in nutritional status.

[25] Roche M, Layrisse M (1966). The nature and causes of “hookworm anemia”.. Am J Trop Med Hyg.

[26] Pawlowski ZS, Schad GA, Stot GJ (1991). Hookworm infection and anaemia. Approaches to prevention and control.

[27] Layrisse M, Roche M, Aparcedo L, Martínez-Torres C (1967). Blood loss due to infection with *Trichuris trichiura*.. Am J Trop Med Hyg.

[28] Ministry of Health (2005). Report on the national survey of current situation of major human parasite diseases in China.

[29] United Nations (2008). World population database: the 2006 revision population database.. http://esa.un.org/unpp/index.asp?panel2.

[30] People's Republic of China (2000). http://www.unescap.org/esid/psis/population/database/chinadata/intro.htm.

[31] Census of India (2003). Provisional population totals: India 2001.

[32] WHO (2008). Life tables for WHO member states.. http://www.who.int/whosis/database/life_tables/life_tables.cfm.

[33] Guyatt HL, Chan MS, Medley GF, Bundy DA (1995). Control of *Ascaris* infection by chemotherapy: which is the most cost-effective option?. Trans R Soc Trop Med Hyg.

[34] Chan L, Bundy DA, Kan SP (1994). Aggregation and predisposition to *Ascaris lumbricoides* and *Trichuris trichiura* at the familial level.. Trans R Soc Trop Med Hyg.

[35] Forrester JE, Golden MH, Scott ME, Bundy DA (1990). Predisposition of individuals and families in Mexico to heavy infection with *Ascaris lumbricoides* and *Trichuris trichiura*.. Trans R Soc Trop Med Hyg.

[36] Haswell-Elkins MR, Elkins DB, Anderson RM (1987). Evidence for predisposition in humans to infection with *Ascaris*, hookworm, *Enterobius* and *Trichuris* in a South Indian fishing community.. Parasitology.

[37] Hall A, Anwar KS, Tomkins AM (1992). Intensity of reinfection with *Ascaris lumbricoides* and its implications for parasite control.. Lancet.

[38] Brooker S, Kabatereine NB, Fleming F, Devlin N (2008). Cost and cost-effectiveness of nationwide school-based helminth control in Uganda: intra-country variation and effects of scaling-up.. Health Policy Plan.

[39] Bundy DA, Horton J, Wong MS, Lewis LL (1992). Control of geohelminths by delivery of targeted chemotherapy through schools.. Trans R Soc Trop Med Hyg.

[40] Miguel E, Kremer M (2004). Worms: identifying impacts on health and education in the presence of treatment externalities.. Econometrica.

[41] Kotze AC, Kopp SR (2008). The potential impact of density dependent fecundity on the use of the faecal egg count reduction test for detecting drug resistance in human hookworms.. PLoS Negl Trop Dis.

[42] Kilama WL, Crompton DWT, Nesheim MC, Pawlowski ZS (1989). Sanitation in the control of ascariasis.. Ascariasis and its prevention and control.

[43] Nock IH, Aken'ova T, Galadima M (2006). Deworming: adding public health education to the equation.. Trends Parasitol.

[44] Horton S, Alderman H, Riveras J (2008). Copenhagen Consensus 2008 Challenge Paper: Hunger and Malnutrition.. http://www.copenhagenconsensus.com/.

[45] Sinuon M, Tsuyuoka R, Socheat D, Montresor A, Palmer K (2005). Financial costs of deworming children in all primary schools in Cambodia.. Trans R Soc Trop Med Hyg.

[46] WHO/UNICEF (2004). How to add deworming to vitamin A distribution.

[47] Alderman H, Konde-Lule J, Sebuliba I, Bundy D, Hall A (2006). Effect on weight gain of routinely giving albendazole to preschool children during “Child Health Days” in Uganda: cluster randomised controlled trial.. BMJ.

[48] Hall A, Horton S (2008). Copenhagen Consensus Best Practices paper: deworming.. http://www.copenhagenconsensus.com/.

[49] Partnership for Child Development (1998). Cost of school-based drug treatment in Tanzania.. Health Policy Plan.

[50] Fiedler JL, Chuko T (2008). The cost of Child Health Days: a case study of Ethiopia's Enhanced Outreach Strategy (EOS).. Health Policy Plan.

