# Supplementary material for: The Costs and Cost-Effectiveness of Mass Treatment for Intestinal Nematode Worm Infections Using Different Treatment Thresholds
Source: PLoS Negl Trop Dis. 2009 Mar 31;3(3):e402. doi: 10.1371/journal.pntd.0000402 (PMC2657832; doi:10.1371/journal.pntd.0000402)
Supplement: Table S1 — Data for each country on the prevalence of infection with any of the main types of intestinal nematode worm, the population aged 0–15 years and the proportion aged 2–14 y, used for the classification of countries in Table 3 and the calculations in Table 4. (0.12 MB DOC) [file pntd.0000402.s001.doc]

| Country | Estimated prevalence of any worm^1^ | Population aged 0 – 15 y (thousands)^2^ | Proportion aged 2 – 14 y^3^ |  | Country | Estimated prevalence of any worm^1^ | Population aged 0 – 15 y (thousands)^2^ | Proportion aged 2 – 14 y^3^ |
| --- | --- | --- | --- | --- | --- | --- | --- | --- |
| Algeria | 1.1 | 9,740 | Nc |  | Mali | 20.4 | 5,541 | 0.790 |
| Angola | 89.9 | 7,471 | 0.787 |  | Mauritania | 8.5 | 1,195 | nc |
| Argentina | 29.6 | 10,237 | 0.800 |  | Mauritius | 25.8 | 303 | 0.800 |
| Bangladesh | 84.2 | 53,927 | 0.798 |  | Mexico | 25.9 | 32,069 | 0.799 |
| Barbados | 30.9 | 55 | 0.800 |  | Micronesia | 82.5 | 172 | 0.799 |
| Benin | 3.9 | 3,753 | Nc |  | Mongolia | 0.0 | 745 | nc |
| Bolivia | 33.7 | 3,498 | 0.798 |  | Morocco | 14.5 | 9,234 | nc |
| Botswana | 40.7 | 653 | 0.796 |  | Mozambique | 49.3 | 9,081 | 0.795 |
| Brazil | 44.3 | 51,994 | 0.800 |  | Myanmar | 79.0 | 13,073 | 0.796 |
| Burkina Faso | 28.1 | 6,431 | 0.791 |  | Namibia | 51.1 | 790 | 0.798 |
| Burundi | 56.1 | 3,547 | 0.792 |  | Nepal | 65.1 | 10,556 | 0.798 |
| Cambodia | 75.5 | 5,245 | 0.794 |  | Nicaragua | 26.5 | 2,069 | 0.799 |
| Cameroon | 81.5 | 7,433 | 0.798 |  | Niger | 20.6 | 6,360 | 0.788 |
| Central African Rep | 51.5 | 1,788 | 0.793 |  | Nigeria | 73.0 | 62,673 | 0.791 |
| Chad | 32.6 | 4,691 | 0.791 |  | Oman | 14.5 | 847 | nc |
| Chile | 32.1 | 4,054 | 0.800 |  | Pakistan | 16.2 | 58,736 | nc |
| China | See Table S2 | 283,771 | 0.800 |  | Panama | 50.8 | 981 | 0.800 |
| Colombia | 48.1 | 13,622 | 0.800 |  | Papua New Guinea | 76.4 | 2,465 | 0.798 |
| Congo | 80.4 | 1,513 | 0.795 |  | Paraguay | 64.3 | 2,115 | 0.800 |
| Congo, DR | 89.3 | 27,712 | 0.795 |  | Peru | 50.0 | 8,665 | 0.800 |
| Costa Rica | 20.9 | 1,227 | 0.800 |  | Philippines | 81.4 | 30,578 | 0.799 |
| Cote d'Ivory | 84.8 | 7,756 | 0.795 |  | Puerto Rico | 17.2 | 866 | nc |
| Dominican Republic | 13.2 | 3,172 | Nc |  | Rwanda | 98.5 | 4,013 | 0.793 |
| Ecuador | 49.4 | 4,259 | 0.800 |  | Samoa | 82.5 | 75 | 0.799 |
| Egypt | 18.4 | 24,287 | Nc |  | Sao Tome & Principe | 95.5 | 63 | 0.796 |
| El Salvador | 66.0 | 2,773 | 0.800 |  | Saudi Arabia | 4.9 | 8,154 | nc |
| Equatorial Guinea | 83.0 | 205 | 0.791 |  | Senegal | 47.1 | 4,967 | 0.795 |
| Eritrea | 0.6 | 1,947 | Nc |  | Sierra Leone | 65.4 | 2,389 | 0.787 |
| Ethiopia | 63.3 | 35,116 | 0.795 |  | Solomon Is. | 82.5 | 191 | 0.798 |
| Fiji | 82.5 | 272 | 0.800 |  | Somalia | 55.0 | 3,615 | 0.795 |
| Gabon | 83.9 | 464 | 0.797 |  | South Africa | 66.8 | 15,396 | 0.798 |
| Ghana | 42.4 | 8,796 | 0.795 |  | Sri Lanka | 58.5 | 4,621 | 0.800 |
| Grenada | 30.9 | 36 | 0.800 |  | St. Lucia | 30.9 | 45 | 0.800 |
| Guatemala | 92.6 | 5,484 | 0.799 |  | St.Vincent & Grenadines^4^ | 29.1 | 35 | 0.800 |
| Guinea | 51.1 | 3,906 | 0.793 |  | Sudan | 26.5 | 15,013 | 0.797 |
| Guinea-Bissau | 30.3 | 758 | 0.791 |  | Suriname | 54.2 | 135 | 0.799 |
| Guyana | 34.0 | 230 | 0.798 |  | Syria | 0.0 | 6,914 | nc |
| Haiti | 66.9 | 3,528 | 0.797 |  | Tanzania | 52.2 | 17,085 | 0.795 |
| Honduras | 71.1 | 2734 | 0.800 |  | Thailand | 56.3 | 13,657 | 0.800 |
| India | See Table S2 | 374,144 | 0.798 |  | The Bahamas | 30.9 | 89 | 0.800 |
| Indonesia | 76.5 | 64,147 | 0.799 |  | The Gambia | 75.8 | 646 | 0.796 |
| Iran | 10.1 | 19,961 | Nc |  | Togo | 79.5 | 2,700 | 0.796 |
| Iraq | 5.2 | 11,606 | Nc |  | Tonga | 82.5 | 37 | 0.800 |
| Jamaica | 58.7 | 850 | 0.799 |  | Trinidad & Tobago | 4.5 | 294 | nc |
| Jordan | 8.3 | 2,062 | Nc |  | Tunisia | 0.0 | 2,631 | nc |
| Kenya | 57.7 | 15,176 | 0.795 |  | Uganda | 52.9 | 14,289 | 0.794 |
| Laos | 60.9 | 2,254 | 0.798 |  | Uruguay | 2.8 | 792 | nc |
| Lebanon | 0.0 | 1,148 | Nc |  | Vanuatu | 82.5 | 86 | 0.799 |
| Liberia | 90.9 | 1,614 | 0.790 |  | Venezuela | 56.7 | 8,371 | 0.800 |
| Libya | 0.0 | 1,795 | Nc |  | Vietnam | 76.8 | 25,198 | 0.800 |
| Madagascar | 74.0 | 8,167 | 0.795 |  | Yemen | 35.8 | 9,678 | 0.797 |
| Malawi | 48.4 | 6,224 | 0.795 |  | Zambia | 62.5 | 5,246 | 0.796 |
| Malaysia | 74.4 | 8,053 | 0.800 |  | Zimbabwe | 67.3 | 5,186 | 0.797 |
| Maldives | 78.0 | 100 | 0.799 |  |  |  |  |  |

nc = not calculated for countries with worm prevalence below 20%, as would not receive deworming under either regime.

^1^ Source is prevalence data for each species of intestinal nematode worm from ref [2]; the calculation of multiple infections is described in text.

^2^ Data from ref [29]

^3^ Proportion calculated using WHO life tables [32] as described in Footnote to Table 4.

^4^ The WHO does not provide life tables for St. Vincent and the Grenadines so the value for the proportion of the population in the 2-14 age range for St. Lucia and Barbados was used i.e. 0.800.
