# Supplementary material for: The Costs and Cost-Effectiveness of Mass Treatment for Intestinal Nematode Worm Infections Using Different Treatment Thresholds
Source: PLoS Negl Trop Dis. 2009 Mar 31;3(3):e402. doi: 10.1371/journal.pntd.0000402 (PMC2657832; doi:10.1371/journal.pntd.0000402)
Supplement: Table S2 — Sub-national data for India (left) and China (right) on the prevalence of infection with any of the main types of intestinal nematode worm, the total population, and the population aged 2–14 y, used for the classification of states and territories of India and provinces, autonomous areas and municipalities of China in Table 3 and for the calculations in Table 4. (0.10 MB DOC) [file pntd.0000402.s002.doc]

| India | Estimated prevalence of any worm^1^ | Total population (thousands)^2^ | Population aged 2 – 14 y (thousands)^3^ |  | China | Estimated prevalence of any worm^4^ | Total population (thousands)^5^ | Proportion aged 2 – 14 y (thousands)^6^ |
| --- | --- | --- | --- | --- | --- | --- | --- | --- |
| Andaman & Nicobar Islands | 51.5 | 360 | 104 |  | Anhui | 19.9 | 59,860 | 11,122 |
| Andhra Pradesh | 72.0 | 75,730 | 22,014 |  | Beijing | 2.8 | 13,820 | 2,568 |
| Arunachal Pradesh | 7.2 | 1,090 | 317 |  | Chongqing | 33.9 | 30,900 | 5,741 |
| Assam | 47.3 | 26,640 | 7,744 |  | Fujian | 30.5 | 34,710 | 6,449 |
| Bihar | 4.9 | 82,880 | 24,093 |  | Giansu | 12.9 | 25,620 | 4,760 |
| Chandigarh | 18.1 | 900 | 262 |  | Guangdong | 16.3 | 86,420 | 16,057 |
| Chhatisgarh | 20.9 | 20,800 | 6,045 |  | Guanxi | 39.6 | 44,890 | 8,341 |
| Dadra & Nagar Haveli | 22.4 | 220 | 64 |  | Guizhou | 46.7 | 35,250 | 6,549 |
| Daman & Diu | 9.1 | 160 | 46 |  | Hainan | 54.7 | 7,870 | 1,462 |
| Delhi | 12.7 | 13,780 | 4,007 |  | Hebei | 1.7 | 67,440 | 12,530 |
| Goa | 23.5 | 1,340 | 391 |  | Heilongjiang | 3.2 | 36,890 | 6,854 |
| Gujarat | 11.8 | 50,600 | 14,709 |  | Henan | 7.9 | 92,560 | 17,198 |
| Haryana | 2.4 | 21,080 | 6,129 |  | Hubei | 30.3 | 60,280 | 11,200 |
| Himachal Pradesh | 14.7 | 6,080 | 1,767 |  | Hunan | 35.3 | 64,600 | 12,003 |
| Jammu & Kashmir | 52.6 | 10,070 | 2,927 |  | Jiangsu | 7.3 | 74,380 | 13,820 |
| Jharkhand | 4.9 | 26,910 | 7,823 |  | Jiangxi | 28.4 | 41,400 | 7,692 |
| Karnataka | 53.8 | 52,730 | 15,330 |  | Jilin | 12.0 | 27,280 | 5,069 |
| Kerala | 57.0 | 31,840 | 9,255 |  | Liaoning | 9.5 | 42,380 | 7,874 |
| Lakshadweep | 64.6 | 60 | 18 |  | Nei-Mongol | 1.2 | 23,760 | 4,415 |
| Madhya Pradesh | 20.9 | 60,390 | 17,554 |  | Ningxia | 5.5 | 5,620 | 1,044 |
| Maharashtra | 9.4 | 96,750 | 28,126 |  | Qinghai | 8.2 | 5,180 | 962 |
| Manipur | 47.3 | 2,390 | 694 |  | Shaanxi | 16.2 | 36,050 | 6,698 |
| Meghalaya | 47.3 | 2,310 | 670 |  | Shandong | 13.2 | 90,790 | 16,869 |
| Mizoram | 16.4 | 890 | 259 |  | Shanghai | 2.9 | 16,740 | 3,110 |
| Nagaland | 47.3 | 1,990 | 578 |  | Shanxi | 2.7 | 32,970 | 6,126 |
| Orissa | 12.5 | 36,710 | 10,671 |  | Sichuan | 39.7 | 42,880 | 7,967 |
| Pondicherry | 12.1 | 970 | 283 |  | Tianjing | 1.8 | 10,010 | 1,860 |
| Punjab | 7.3 | 24,290 | 7,061 |  | Xinjiang | 0.7 | 19,250 | 3,577 |
| Rajasthan | 4.9 | 56,470 | 16,417 |  | Xizang/Tibet | 6.5 | 2,620 | 487 |
| Sikkim | 36.4 | 540 | 157 |  | Yunnan | 21.7 | 42,880 | 7,967 |
| Tamil Nadu | 21.2 | 62,110 | 18,056 |  | Zhejiang | 5.7 | 46,770 | 8,690 |
| Tripura | 44.3 | 3,190 | 928 |  |  |  |  |  |
| Uttar Pradesh | 19.8 | 166,050 | 48,272 |  |  |  |  |  |
| Uttaranchal | 19.8 | 8,480 | 2,465 |  |  |  |  |  |
| West Bengal | 33.1 | 80,220 | 23,320 |  |  |  |  |  |
|  |  |  |  |  |  |  |  |  |

^1^ Prevalence data for each species of intestinal nematode worm from ref [2]; the calculation of multiple infections is described in the text. Data for six states were not available: three of these were newly created states (Jharkand, Chhatisgarh and Uttaranchal) so the data from the “parent” state were used (Bihar, Madhya Pradesh and Uttar Pradesh respectively); and three were small states in the north-east (Manipur, Meghalaya and Nagaland) for which data for a larger, adjacent state was used (Assam).

^2^ Data from the 2001 Census of India [31]

^3^ Calculation based on a proportion of 0.2907 from UN population database [29] and WHO life tables [32]

^4^ Published data on combined prevalence of infection from ref [28]

^5^ Data from the 2000 Census of China [30]

^6^ Calculation based on a proportion of 0.1858 from UN population database [29] and WHO life tables [32]
